# Effects of Audible Sound and Ultrasound on Microalgae: Growth Responses and Metabolite Production

**DOI:** 10.3390/biology15161340

**Published:** 2026-08-07

**Authors:** Eleftherios Touloupakis, Raffaella Margherita Zampieri, Cecilia Faraloni, Mario Pagano

**Affiliations:** 1 Research Institute on Terrestrial Ecosystems, National Research Council, Via Madonna del Piano 10, 50019 Sesto Fiorentino, Italy; raffaellamargherita.zampieri@unifi.it; 2 Department of Agriculture, Food, Environment and Forestry, University of Florence, Via San Bonaventura 13, 50145 Firenze, Italy; 3 Institute of Bioeconomy, National Research Council, Via Madonna del Piano 10, 50019 Sesto Fiorentino, Italy; cecilia.faraloni@cnr.it

**Keywords:** microalgae, sound waves, ultrasound, acoustic stimulation, biotechnology

## Abstract

Developing sustainable strategies to enhance microalgal productivity is a major challenge in modern biotechnology. Beyond optimizing light, nutrient supply, and temperature, physical stimulation methods have recently attracted growing interest. Among these, audible sound and ultrasound have emerged as promising tools capable of influencing growth, biomass accumulation, and metabolite production in several microalgal species. Although the available literature is still limited, current evidence suggests that acoustic stimulation can alter cellular physiology through mechanosensitive responses, changes in membrane permeability, and modulation of metabolic activity. This review summarizes current knowledge on the effects of sound waves on microalgae, discusses potential mechanisms involved in acoustic perception, and highlights future research directions.

## Simple Summary

Microalgae have great potential as a sustainable source of biofuels and high-value compounds, but large-scale production remains challenging. Sound-based technologies, including audible sound and ultrasound, have recently emerged as innovative tools to enhance microalgal growth and productivity without the use of chemicals. This review examines the effects of acoustic stimulation on microalgae, highlights the possible mechanisms underlying these responses, and identifies key research needs for future applications in biotechnology.

## 1. Introduction

Microalgae are considered among the most promising biological resources to produce biofuels, pigments, antioxidants, nutraceuticals, and pharmaceuticals [1,2,3,4,5,6]. However, large-scale cultivation remains constrained by high production costs and relatively low biomass productivity [7]. Consequently, considerable efforts have been focused on developing strategies to enhance biomass yields and metabolite accumulation. Various cultivation approaches have been explored to address these challenges, including the optimization of nutrient regimes, carbon supply, light conditions, and cultivation system design [7,8]. Substantial progress has been made in the development of photobioreactor (PBR) technologies [9]. Modern PBRs are highly engineered cultivation systems designed to maximize light utilization, improve mass transfer, and ensure precise control of environmental parameters.

The accumulation of high-value metabolites in microalgae strongly depends on species-specific characteristics, genetic variability, and cultivation conditions [10]. Factors such as light intensity, temperature, and nutrient availability can substantially alter metabolic fluxes, often promoting the synthesis of compounds involved in stress protection defense [11]. Experimental evidence indicates that environmental conditions regulate not only growth but also lipid, protein, and pigment biosynthesis, thereby influencing both biomass quality and product yields [7,12]. As a result, increasing efforts have been directed toward understanding how environmental conditions regulate metabolism and influence product yield and quality.

Microalgae are not isolated organisms but actively interact through the production, release, and perception of chemical signals, exchanging extracellular molecules within and between species [13]. These signals regulate key physiological and ecological processes, including growth, cell-cycle progression, nutrient acquisition, stress responses, and bloom dynamics [14]. Microalgae can detect and respond to signals released by conspecifics and to compounds produced by other species, thereby influencing community composition and ecosystem functioning [15]. Chemical signaling therefore represents a fundamental mechanism through which microalgae coordinate their behavior, interact with neighboring organisms, and contribute to the assembly and stability of aquatic microbial communities [16]. While microalgal communication has traditionally been associated with chemical signaling mediated by infochemicals and allelochemicals, other forms of communication remain poorly understood, and the perception of sound in microalgae is still largely unexplored [16].

Sound is a mechanical wave produced by the propagation of pressure fluctuations through a material medium, such as air, water, or solids. Sound is a promising physical tool for optimizing biotechnological processes, owing to its cost-effectiveness and environmentally friendly nature. Sound wave technology has been applied to a variety of different species of plants, fungi and microalgae, with growth responses being strongly influenced by acoustic parameters such as frequency, sound pressure level, exposure duration, and distance from the sound source [17,18,19,20]. Studies conducted in higher plants, fungi, and other microorganisms have shown that acoustic stimulation can influence growth, metabolism, stress responses, and cellular signaling pathways, providing a broader biological framework for understanding acoustic responses in microalgae [17,18,19,20]. Experimental studies have shown that acoustic stimulation can promote growth and biomass productivity in several microalgal species, including *Chlorella pyrenoidosa*, *Picochlorum oklahomensis*, and *Haematococcus pluvialis* [21,22,23].

Ultrasound refers to high-frequency acoustic waves beyond the range of human hearing and is widely used in medical, industrial, and biotechnological applications because of its ability to induce physical and chemical effects within a medium [24]. When transmitted through liquids, ultrasound can generate cavitation, characterized by the formation, growth, and collapse of microbubbles [24]. The resulting localized shear forces, microstreaming, and transient increases in temperature and pressure can significantly affect mass transfer, cell permeability, and biochemical reactions [24]. These properties have attracted growing interest in microalgal biotechnology, where ultrasound has been investigated as a tool to enhance cell growth, metabolite production, biomass harvesting, and lipid extraction [25,26,27,28].

Emerging evidence suggests that acoustic phenomena may play a role in microalgal interactions. Freeman et al. [29] reported that photosynthetic activity in marine algae generates sound through the formation and release of oxygen bubbles, demonstrating that these organisms produce acoustic emissions within the 2–20 kHz frequency range. Byeon et al. [30] evaluated the effects of pile-driving noise on marine microalgae and found significant alterations in cell morphology and physiological activity. Reductions in cell size, granularity, and esterase activity were observed, demonstrating species-specific sensitivity to acoustic stress and underscoring the need for multiple indicators to assess noise-related impacts on microalgae.

**Figure 1:**
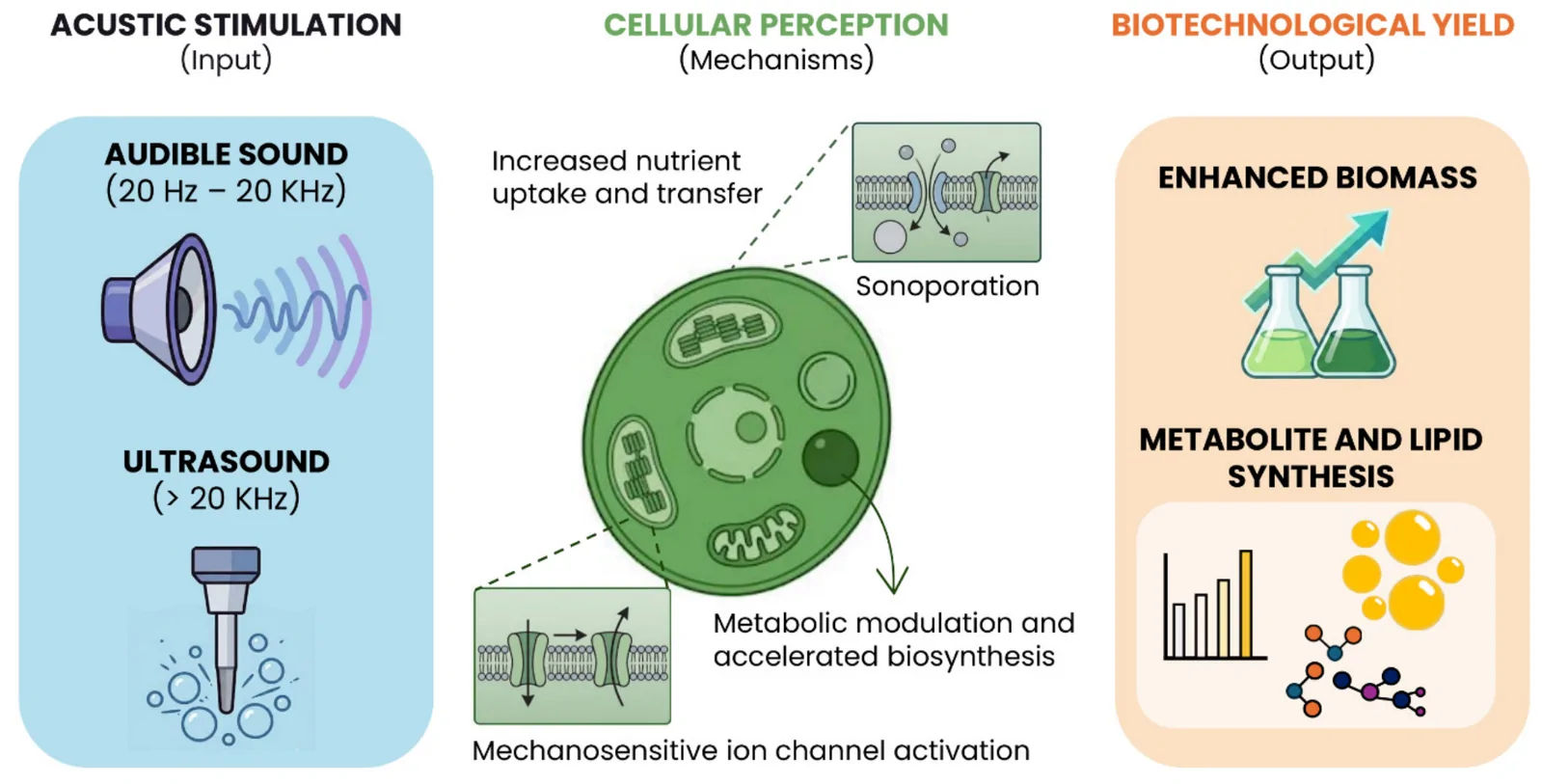
Schematic representation of the effects of acoustic stimulation on microalgae. Audible sound and ultrasound trigger cellular responses through mechanosensitive ion channels and sonoporation, enhancing nutrient uptake and metabolic activity. These processes ultimately lead to increased biomass production and enhanced synthesis of lipids and other high-value metabolites for biotechnological applications.

Microalgal cells can respond by changing cell division, dimensions, signal transduction, gene expression, and membrane ion channel activation to mechanical stimuli, hydrostatic pressure, and changes in plasma-membrane tension, and to sound-induced vibrations [29]. Figure 1 summarizes the main mechanisms through which audible sound and ultrasound affect microalgal cells and their potential biotechnological outcomes. Golub and Levtun [31] investigated how acoustic stimulation at different frequencies affects the metabolism, biomass production, and lipid synthesis of *Chlorella vulgaris*. Exposure to sound waves at 10 and 15 kHz enhanced pigment biosynthesis but inhibited overall biosynthetic activity, reducing biomass growth while promoting triacylglycerol accumulation. In contrast, ultrasound treatment at 20 kHz stimulated the active transport of ions and metabolites across the cell membrane, enhanced cellular metabolism, and accelerated biosynthetic processes, resulting in a 10% increase in biomass production and a threefold increase in lipid synthesis.

Despite growing interest in acoustic stimulation as a non-invasive strategy to improve microalgal cultivation by promoting biomass productivity and metabolite accumulation, the underlying mechanisms governing these responses remain only partially understood. Existing studies report promising outcomes in terms of enhanced growth, metabolic regulation, and lipid accumulation, yet the mechanisms involved and their scalability remain largely unresolved. While previous reviews have addressed specific aspects of acoustic stimulation in biological systems, this review presents a dedicated overview of the effects of both audible sound and ultrasound on microalgae. It places particular emphasis on their influence on growth, physiology, and metabolite production, as well as on cultivation system design, acoustic propagation, current research gaps, and future perspectives.

This mini-review therefore provides an overview of the current state of the art regarding sound- and ultrasound-induced responses in microalgae, with a focus on growth performance, metabolite production, and potential applications in PBR systems. Current limitations and emerging research priorities are also discussed to guide future developments in this rapidly evolving field.

## 2. Effects of Sound and Ultrasound on Microalgal Growth

### 2.1. Sound

Acoustic stimulation has emerged as a noninvasive, potentially low-cost technology. Although acoustic responses have been extensively investigated in higher plants, studies on microalgae are more recent and remain relatively fragmented. Nevertheless, available evidence suggests that specific sound frequencies can positively influence microalgal growth and metabolism. Depending on its frequency, sound can be classified as audible (20 Hz–20 kHz), infrasonic (<20 Hz), or ultrasonic (>20 kHz). The hertz (Hz) is the SI unit used to quantify frequency, defined as the number of complete oscillatory cycles occurring per unit time; thus, 1 Hz is equivalent to 1 cycle s^−1^. In acoustics, sound pressure levels are represented on a logarithmic scale, whereby an increment of 1 bel denotes a tenfold increase in the acoustic energy transported by the wave. Owing to the large magnitude of the bel for practical measurements, the decibel (dB), defined as one-tenth of a bel, is conventionally used to express acoustic levels. Acoustic stimulation studies in microalgae have primarily focused on audible sound and ultrasound, whereas the effects of infrasonic frequencies (<20 Hz) have received considerably less attention. Acoustic waves can be classified into three main categories based on their frequency range and biological applications, as summarized in Table 1.

Jiang et al. [21] studied the effects of sound waves on *Chlorella pyrenoidosa* to identify the optimal frequency for promoting growth. Their experiments showed that sound waves at 0.4 kHz significantly enhanced the growth of *C. pyrenoidosa*, resulting in a 12–30% increase compared with the control. Ganguli et al. [32] studied the impact of audible sound on the growth and physiology of *Microcystis* sp., *Arthrospira* sp., *Chlorococcum* sp., and *Cladophora* sp. They observed positive effects on growth and biochemical parameters, confirming that audible sound can influence microalgal physiology. Cai et al. [22] reported that growth stimulation of *Picochlorum oklahomensis* was greatest at a sound frequency of 2.2 kHz. In addition, sound-exposed cultures reached the steady-state growth phase more rapidly than control cultures, demonstrating the potential of sound stimulation to enhance microalgal biomass production. This finding is particularly noteworthy because 2.2 kHz is a predominant frequency component of many natural sounds [22]. Christwardana and Hadiyanto [23] studied the effect of sound on the growth and productivity of *Haematococcus pluvialis*. They exposed microalgal cultures to audible sound at an intensity of 60 dB for 8 and 22 days and found that sound exposure increased the growth rate by 58%.

Sound can be exploited to synthesize microalgal cellular components of significant biotechnological relevance. The application of sound to microalgae currently covers only a small area that clearly deserves further investigation. The frequencies and intensities that promote growth and productivity vary between species, and those explored so far are not sufficient to provide a complete picture of the possible combinations. Collectively, these studies indicate that responses are highly dependent on species, frequency, intensity and exposure duration.

Despite the generally positive effects reported in the literature, responses to audible sound are not always consistent across species or cultivation conditions. The limited number of available studies, combined with differences in acoustic parameters and experimental designs, makes direct comparison difficult. Moreover, frequencies that stimulate growth in one species may produce neutral or even inhibitory effects in another, indicating that acoustic responses are highly species-specific and context-dependent.

Rusyn [33] recently presented a comprehensive analysis of the use of sound treatment in microalgal cultivation to enhance productivity and lipid yields in the context of biodiesel production. Representative examples of sound-induced effects on microalgal growth are summarized in Table 2.

### 2.2. Ultrasound

Ultrasound is characterized by frequencies above 20 kHz and may induce effects through cavitation and localized mechanical stresses. Several hypotheses have been proposed regarding the effects of ultrasound on cells. Lentacker et al. [24] described the mechanisms by which ultrasound interacts with cells, highlighting its ability to induce transient pores in the cell membrane and thereby increase membrane permeability. This phenomenon, known as sonoporation, enhances mass transfer by facilitating substrate uptake and metabolite exchange, thereby improving bioconversion efficiency [24].

Beyond growth stimulation, ultrasound has also been explored as a tool for recovering intracellular compounds. In parallel, the concept of “microalgal milking” has emerged as a promising strategy to extract high-value metabolites while preserving cell viability and enabling repeated cultivation cycles. In *Haematococcus pluvialis*, the recovery of astaxanthin through mild extraction approaches has been proposed to improve process sustainability and reduce biomass loss [38]. Ultrasound-assisted extraction has also been investigated as an effective method for enhancing astaxanthin recovery from *Haematococcus pluvialis* biomass [39].

Dvoretsky et al. [25] demonstrated that ultrasonic treatment can stimulate *Chlorella* growth and enhance intracellular metabolite accumulation. These effects are associated with increased metabolic activity driven by a rise in temperature and the removal of extracellular inhibitory phenolic compounds, resulting in more productive biomass. Pereira et al. [40] investigated the effects of ultrasound on *Chlorella zofingiensis* growth and lipid accumulation, finding that ultrasound treatment increased biomass concentration but decreased lipid content. Ultrasound in microalgal cultivation can be combined with other physical or chemical factors to further enhance biomass productivity and metabolite accumulation. In this context, Kan et al. [41] demonstrated that the combined application of ultrasound and melatonin synergistically promoted the growth of *Dunaliella salina,* resulting in increased cell density, photosynthetic pigment production, and lipid accumulation. Pham et al. [42] investigated the effects of audible sound stimulation on the performance of *Chlorella* sp. in traditional market wastewater treatment and demonstrated its potential to enhance total nitrogen removal efficiency and improve microalgal-based nutrient remediation.

Han et al. [43] reported that ultrasonic treatment applied at the end of the exponential growth phase significantly enhanced lipid accumulation in *Scenedesmus quadricauda* without adversely affecting biomass productivity. Under optimized conditions, lipid content reached 57.5% of the dry cell weight, highlighting the potential of ultrasound as an effective strategy to improve microalgal lipid production. The same group demonstrated that appropriate ultrasound treatment effectively enhanced lipid accumulation in *Anabaena variabilis* without adversely affecting biomass production, protein content, or fatty acid composition [43]. Under optimized conditions, lipid content and productivity increased substantially.

Xie et al. [44] showed that combining seawater and ultrasound significantly improved the cultivation performance of *Chlorella sorokiniana*, resulting in increased biomass production and lipid accumulation. Sivaramakrishnan and Incharoensakdi [27] reported that optimized ultrasound treatment significantly enhanced both biomass production and lipid accumulation in *Scenedesmus* sp. In addition to increasing total lipid productivity, ultrasound stimulation improved photosynthetic performance, as evidenced by higher chlorophyll *a* and carotenoid contents, as well as enhanced oxygen evolution rates, ultimately promoting overall microalgal productivity. Han et al. [45] demonstrated that ultrasonic treatment significantly improved the growth performance of *Haematococcus lacustris* by increasing cell density, chlorophyll content, and the expression of genes associated with cell proliferation and nitrogen assimilation.

In their recent study, Zhang et al. [46] investigated the use of low-intensity ultrasound to improve the stability of microalgae–bacteria consortia and promote lipid accumulation. In this context, stability refers to maintaining balanced microalgae–bacteria interactions and sustained culture performance over time. Low-intensity ultrasonic stimulation enhanced culture performance by increasing culture homogeneity and fostering more effective interactions between cells and the surrounding medium. These changes improved nutrient availability and supported metabolic processes associated with photosynthetic efficiency, leading to greater lipid storage within the biomass. In addition, ultrasound induced a metabolic shift that favored lipid production over other biosynthetic pathways while providing a more energy-efficient mixing approach than conventional agitation methods.

Ren et al. [47] demonstrated that optimized low-intensity ultrasound enhanced biomass production and lipid accumulation in the microalgal mutant *Scenedesmus* sp. Z-4 by improving membrane permeability, nutrient transport, and photosynthetic activity. However, they also reported that excessive ultrasonic exposure inhibited both biomass production and lipid accumulation, highlighting the strong dependence of microalgal responses on treatment intensity and exposure conditions [47].

Although many studies have reported beneficial effects of low-intensity ultrasound, excessive acoustic exposure may negatively affect microalgal performance. High ultrasound intensity or prolonged treatment can induce excessive cavitation, leading to altered membrane integrity, cellular stress, and reduced physiological performance [24]. These observations highlight the narrow operational window between beneficial stimulation and detrimental cellular effects and emphasize the need for careful optimization of treatment parameters. Several studies have reported that the timing and duration of ultrasound application are critical determinants of the final response, with treatments applied during specific growth stages often producing different outcomes than continuous exposure regimes. Representative examples of ultrasound-induced effects on microalgal growth are summarized in Table 3.

### 2.3. Putative Mechanisms of Acoustic Stimulation in Microalgae

Although the mechanisms underlying acoustic responses in microalgae remain incompletely understood, several hypotheses have been proposed. Audible sound may act as a mechanical stimulus that influences membrane properties, ion transport, and intracellular signaling pathways [17,19]. These effects could alter cellular metabolism and photosynthetic activity, ultimately affecting growth and metabolite accumulation [22,23,31,35]. In contrast, ultrasound exerts additional physical effects through cavitation, microstreaming, and transient membrane permeabilization, which can enhance mass transfer and nutrient uptake [24,50]. These processes may facilitate metabolite transport and stimulate physiological responses associated with biomass production and lipid accumulation [25,26,43,47]. However, the relative contributions of these mechanisms remain unclear and are likely influenced by species-specific characteristics, acoustic parameters, and cultivation conditions [33,51,52,53]. Further molecular and systems biology studies are needed to elucidate the pathways involved in responses to acoustic stimulation.

## 3. Photobioreactors and Acoustic Stimulations

In recent years, sound stimulation has emerged as a promising strategy for improving microalgal cultivation in PBRs. The effectiveness of acoustic treatments depends not only on biological responses but also on the physical propagation of sound waves within liquid cultivation systems. Therefore, understanding both acoustic transmission and bioreactor design is essential for developing scalable applications.

### 3.1. Acoustic Wave Propagation in Liquid Cultures and Photobioreactors

Sound waves generally propagate more efficiently in liquids than in air because of the higher density and lower compressibility of aqueous media. However, acoustic energy distribution within microalgal cultures may be strongly influenced by bioreactor geometry, culture depth, cell concentration, aeration rate, mixing conditions, and the distance between the acoustic source and the culture [37,51,52]. As sound waves propagate through a medium, their intensity progressively decreases due to geometric spreading and energy dissipation. Consequently, cultures located closer to the sound source may receive stronger acoustic stimulation than those positioned farther away [37,54]. This factor is particularly important in large-scale cultivation systems, where maintaining a uniform acoustic field throughout the reactor volume can represent a significant engineering challenge.

In laboratory-scale cultivation systems, where culture volumes are relatively small and acoustic sources are positioned close to the vessel, sound energy can be distributed more uniformly throughout the culture. As bioreactor size increases, however, acoustic propagation becomes more complex. Reflection, attenuation, refraction, and scattering can produce spatial heterogeneity in the acoustic field, resulting in different levels of stimulation within the same cultivation system [51,52,54]. Gas bubbles generated by aeration can further alter sound transmission by absorbing and scattering acoustic energy, thereby affecting wave propagation and energy distribution [54].

In ultrasound applications, cavitation is considered one of the main mechanisms underlying biological responses. The formation, oscillation, and collapse of microbubbles generate localized shear forces, microstreaming, and transient pressure gradients that can increase membrane permeability and enhance mass transfer [24,50]. These phenomena may improve nutrient uptake and metabolite exchange, but excessive cavitation intensity can also cause cellular damage and reduce culture performance [24,28,54].

PBR configuration is another critical factor influencing acoustic treatment efficiency. Tubular, flat-panel, bubble-column, and airlift PBRs differ substantially in geometry, hydrodynamics, gas–liquid interactions, and wall materials, all of which can affect acoustic wave propagation and energy distribution within the culture [35,51]. Consequently, identical acoustic treatments may induce different physiological responses depending on the cultivation system employed. Despite the growing interest in acoustic stimulation, relatively few studies have quantitatively characterized acoustic field distribution in microalgal cultivation systems, particularly under pilot- and industrial-scale conditions [51,53].

Future investigations should integrate biological assessments with acoustic characterization of cultivation systems to better resolve energy distribution patterns and identify scalable operating conditions. The combined use of acoustic modeling, computational fluid dynamics, and experimental measurements may facilitate the development of optimized PBRs capable of delivering reproducible and energy-efficient acoustic stimulation [51,52,53].

### 3.2. Applications of Acoustic Stimulation in Photobioreactors

Acuna-Gonzalez et al. [51] characterized acoustic wave propagation within a bioreactor under both empty and liquid-filled conditions to evaluate the system’s ability to generate reproducible frequency patterns. Their results showed that acoustic stimulation can be effectively implemented in bioreactors at pilot and industrial scales, supporting its potential as a noninvasive strategy to regulate biomass production and metabolite synthesis. Tambunan et al. [37] investigated the effects of audible sound on the growth of *Chlorella* DPK-01 cultivated in a tubular PBR. They observed a higher growth rate and shorter doubling time in *Chlorella* DPK-01 cultures exposed to the sound source at a shorter distance, suggesting that audible sound with certain frequencies and an appropriate exposure method may increase *Chlorella* growth. Lakshmi Narasimhan et al. [55] investigated ultrasound pretreatment as a strategy to overcome dormancy and enhance the productivity of *Haematococcus lacustris*. Ultrasonically treated cysts showed significantly higher germination rates, faster cell proliferation, and increased astaxanthin accumulation in both flask cultures and bubble-column PBRs. Ultrasound exposure improved intracellular astaxanthin content and volumetric productivity, reaching increases of up to 36.9% and 57.4%, respectively, demonstrating its potential to enhance microalgal cultivation performance. Tabernero et al. [28] recently reviewed the role of ultrasound technology in enhancing the sustainability and efficiency of microalgae-based biofuel production. They highlighted that ultrasonic treatment applied during the stationary growth stage can promote lipid accumulation, while induced alterations in cell structures facilitate biomass recovery and improve the extraction of intracellular lipids. Ortenzi et al. [53] proposed the design and development of a Sono-PBR, a modular, adaptable cultivation system capable of delivering ultrasound irradiation during microalgal growth. Using *Chlorella vulgaris* and *Desmodesmus* sp. as model organisms, the authors evaluated different ultrasound treatments to identify operating conditions that promote either biomass accumulation or the production of valuable bioproducts [53]. The development of the Sono-PBR also demonstrates the growing interest in translating acoustic stimulation technologies toward industrial applications. Notably, the system was developed in collaboration with the innovative startup Yeastime, highlighting the potential for scalable ultrasound-assisted cultivation platforms for microalgal production [53].

The beneficial effects of ultrasound appear to depend strongly on treatment intensity. Excessive exposure may cause cellular damage, whereas moderate stimulation may enhance nutrient transport and metabolic activity. Although both audible sound and ultrasound can positively influence microalgal growth and metabolite production, they differ substantially in their frequency ranges, mechanisms of action, and physiological effects. A comparative overview is presented in Table 4.

## 4. Current Limitations and Research Gaps

Despite the encouraging results reported to date, the application of sound and ultrasound in microalgal biotechnology remains at an exploratory stage. Most investigations have focused on a limited number of species, constraining the generalization of observed responses. Moreover, substantial variability in experimental conditions, such as acoustic frequency, intensity, exposure duration, and cultivation system design, hampers direct comparison among studies and limits the identification of optimal treatment parameters.

Another factor that merits greater attention is the acoustic exposure regime. In the available studies, sound and ultrasound treatments have been applied with markedly different exposure durations, application frequencies, and treatment schedules. Some investigations used continuous stimulation, whereas others applied intermittent or stage-specific treatments [23,43,45,47]. These differences may substantially influence microalgal responses and could partly explain the variability observed among studies. Future research should therefore systematically evaluate exposure duration, duty cycles, treatment frequency, and the timing of acoustic stimulation relative to growth phase to establish reproducible and scalable protocols.

A further research gap concerns the limited understanding of acoustic wave propagation under realistic cultivation conditions. While most available studies have been carried out at laboratory scale, information on acoustic energy distribution, attenuation, and frequency-dependent transmission in pilot- and industrial-scale PBRs remains scarce [51,53]. Future work should combine biological analyses with acoustic characterization and modeling approaches to enable the rational design of scalable, sound-based cultivation systems.

Unlike audible sound and ultrasound, the potential applications of infrasonic waves (<20 Hz) in microalgal biotechnology remain largely unexplored. Another important limitation is the scarcity of mechanistic studies at the molecular level. Transcriptomic, proteomic, and metabolomic investigations remain limited, and no specific acoustic receptors have yet been identified in microalgae. As a result, the cellular and molecular mechanisms underlying acoustic responses are still poorly understood. Future research should focus on standardized protocols, broader taxonomic coverage, and a clearer understanding of the mechanisms underlying acoustic responses in microalgae [28,33].

Recent studies suggest that acoustic stimulation can be further enhanced when combined with other cultivation strategies and physical treatments. For example, ultrasound has been applied in combination with nutrient stress, salinity stress, algae-bacteria consortia, and bioelectrochemical approaches to further stimulate metabolite accumulation and lipid production [33,44,46,49]. Similarly, integrating acoustic treatments with advanced cultivation and reactor optimization strategies may generate synergistic responses that exceed those achieved by individual treatments alone. Nevertheless, the available evidence remains limited, and further studies are required to evaluate the effectiveness, reproducibility, and scalability of combined approaches under different cultivation conditions.

**Figure 2:**
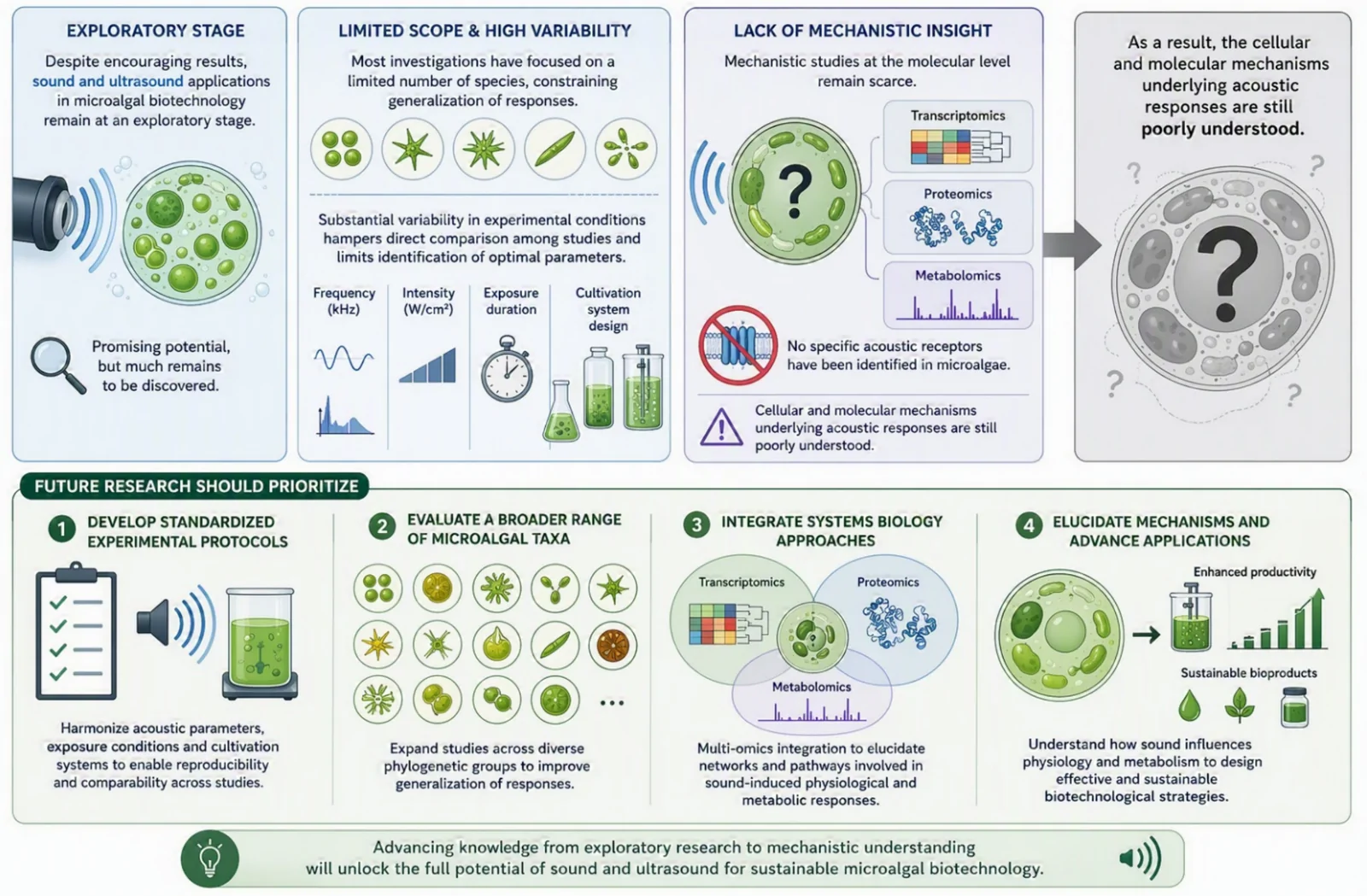
Current limitations and future research priorities for the application of sound and ultrasound in microalgal biotechnology.

A schematic overview of the main limitations and future research directions related to acoustic stimulation in microalgal biotechnology is presented in Figure 2.

**Figure 3:**
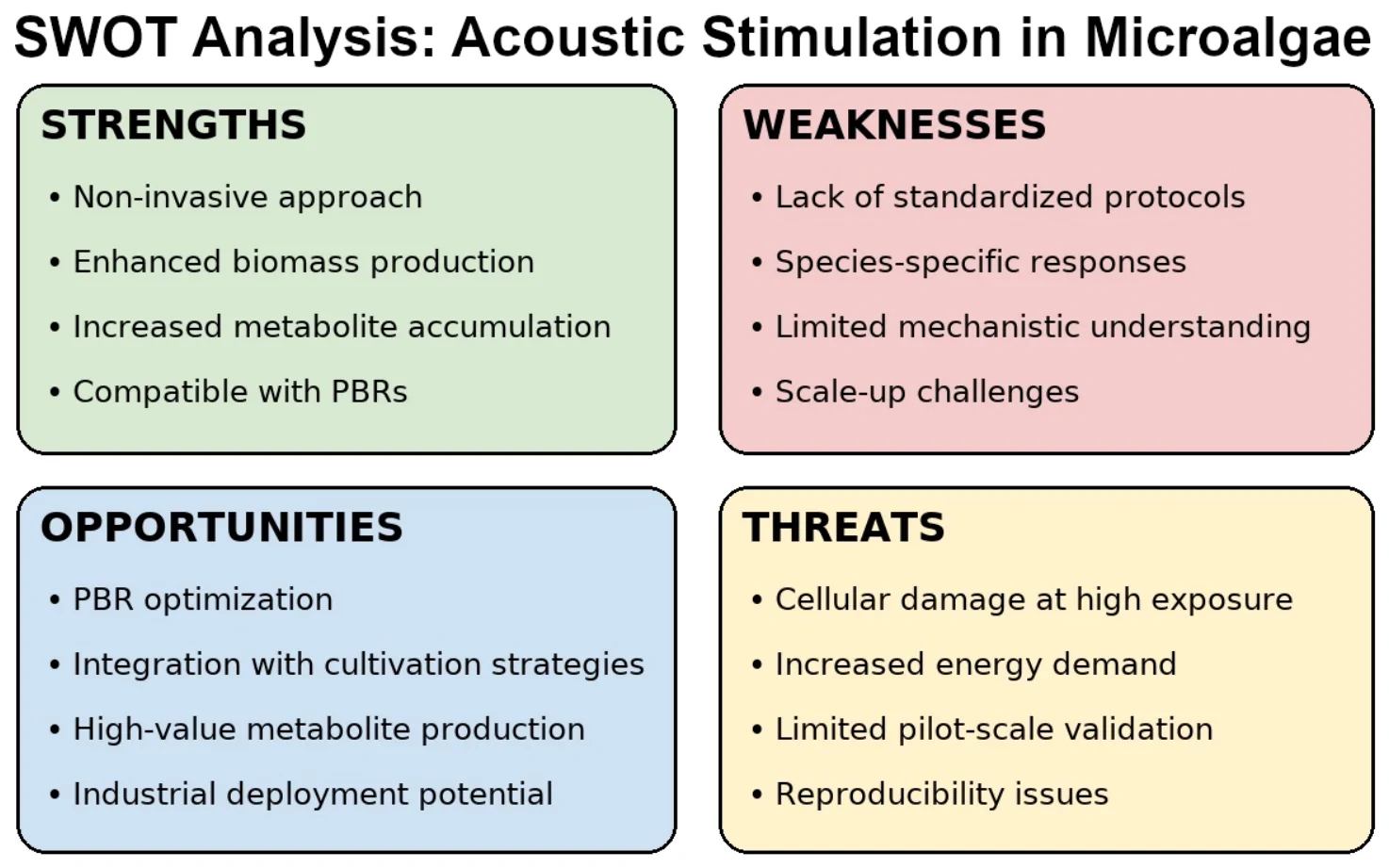
SWOT analysis of audible sound and ultrasound applications in microalgal biotechnology.

The current status of acoustic stimulation technologies in microalgal biotechnology can be summarized through a SWOT (Strengths, Weaknesses, Opportunities, and Threats) analysis (Figure 3).

## 5. Conclusions

Current evidence suggests that acoustic stimulation can positively influence microalgal growth, biomass production, and metabolite accumulation. Both audible sound and low-intensity ultrasound can enhance physiological performance under appropriate conditions. However, responses are highly species-specific and strongly dependent on stimulation parameters. Although the underlying mechanisms remain incompletely understood, altered membrane transport and modulation of metabolic activity are likely involved. Further research integrating physiology, molecular biology, and PBR engineering will be necessary before acoustic technologies can be reliably implemented in industrial microalgal cultivation.

## Figures and Tables

**Table 1:** Classification of acoustic waves and their general applications in biological systems.

Sound Type	Frequency Range	General Applications
Infrasound	<20 Hz	Limited biological applications; effects on microalgae remain largely unexplored
Audible sound	20 Hz–20 kHz	Stimulation of growth, metabolism, photosynthetic activity, and metabolite production in plants and microalgae
Ultrasound	>20 kHz	Enhancement of mass transfer, sonoporation, metabolite production, biomass harvesting, and extraction processes

**Table 2:** Sound-induced effects during microalgal growth.

Microalgal Strain	Sound Treatment	Effects	Ref.
*H. pluvialis* strain UTEX 2505	Music treatment, average frequency of “Blues for Elle” 0.28 kHz, 60 dB, played 8 h per day for 22 days	Increased growth rate by 58%	[23]
*Synechococcus* HS-9	Sine wave, 279.9 Hz, applied for 3 h per day for 18 days, tubular PBR 500 mL	Higher growth and lipid content by 17%	[34]
*Dunaliella salina*	Sound treatment, 200 Hz, bubble column PBR with a working volume of 1 L	Increased biomass up to 50%	[35]
*Picochlorum oklahomensis* UTEX B 2795	Sound waves, 2200 Hz, sound pressure level 90 ± 2 dB, 5 L glass bottles	Increased biomass production and oil content by 27.5%	[22]
*Chlorella* DPK-01	Sine soundwave, 279.9 Hz, exposure in 630 mL tubular PBR cultivated in NPK fertilizer	Higher cell density and higher growth rate	[36]
*Chlorella* DPK-01	Sine soundwave, 279.9 Hz, applied under light exposure, 630 mL tubular PBR	Increased lipid production	[37]
*Chlorella pyrenoidosa*	Sound wave, 400 Hz, 80 dB, applied for 3 h, 3 L flasks	Improved growth rate by 12–30%	[21]

**Table 3:** Ultrasound-induced effects during microalgal growth.

Microalgal Strain	Ultrasound Conditions	Effects	Reference
*Chlorella ellipsoidea* TISTR 8260	50 kHz, applied for 1 min, 2 L flasks	Biomass increase 10%	[48]
*Chlorella sorokiniana* SDEC-18	100 W, applied for 10 min, coupled seawater stress, 1 L flasks	Lipid increase 220%	[44]
*Desmodesmus intermedius* Z_8_	40 kHz, 500 W, applied for 10 min, coupled nitrogen and phosphorus stress, 0.3 L conical flasks	Lipid increase 21%	[49]
*Nannochloris* sp. 424–1	26 kHz, 1 W, applied for 1–3 min per day, 0.2 L flasks	Biomass increase 45% Lipid increase 38%	[12]
*Scenedesmus* sp. Z4	20 kHz, 20 W, applied for 2 s per day for 6 days, 0.24 L reactors	Biomass increase 26.9%, Lipid increase 37%	[47]
*Scenedesmus* sp.	20 W, applied for 4 min with 2 s interval	Biomass increase 40% Lipid increase 70%	[27]
*Scenedesmus quadricauda*	40 kHz, 200 W, applied for 20 min, 0.3 L flasks	Biomass increase 30%, Lipid increase 57.5%	[26]

**Table 4:** Comparison between audible sound and ultrasound applications in microalgal cultivation.

Parameter	Audible Sound	Ultrasound
Frequency range	20 Hz–20 kHz	>20 kHz
Primary mechanism	Mechanical stimulation and cellular signaling	Cavitation, microstreaming, sonoporation
Main physiological effects	Enhanced growth, photosynthesis, and metabolite production	Enhanced mass transfer, nutrient uptake, growth, and lipid accumulation
Biomass productivity	Frequently increased under optimal conditions	Frequently increased under controlled exposure conditions
Metabolite production	Increased pigments and lipids reported in some species	Enhanced lipid and high-value metabolite accumulation commonly reported
Risk of cellular damage	Generally low	Possible under excessive intensity or exposure time
Scale-up feasibility	Relatively simple implementation	More complex due to cavitation control and energy distribution
Main limitations	Lack of protocol standardization and species-specific responses	Potential cell damage and scale-up challenges

## Data Availability

No new data were created or analyzed in this study. Data sharing is not applicable to this article.
